# Applying Multi-Theory Model (MTM) of Health Behavior Change to Predict Water Consumption Instead of Sugar-Sweetened Beverages

**Published:** 2017-02-25

**Authors:** Manoj Sharma, Hannah Priest Catalano, Vinayak K. Nahar, Vimala C. Lingam, Paul Johnson, M. Allison Ford

**Affiliations:** ^1^ Behavioral & Environmental Health, School of Public Health, Jackson State University, Jackson, MS, USA; ^2^ Public Health Studies, School of Health and Applied Human Sciences, University of North Carolina Wilmington, Wilmington, NC, USA; ^3^ Department of Health, Physical Education, and Exercise Science, Lincoln Memorial University, Harrogate, TN, USA; ^4^ Department of Management, School of Business Administration, University of Mississippi, MS, USA; ^5^ Department of Health, Exercise Science & Recreation Management, School of Applied Sciences, University of Mississippi, MS, USA

**Keywords:** Water consumption, Water intake, Health behavior, Theoretical model

## Abstract

**Background:** A substantial proportion of college students to not drink enough water and consume sugar-sweetened beverages (SSBs). Consumption of SSBs is associated with weight gain, obesity, type 2 diabetes mellitus, dental carries, and increased risk for cardiovascular disease. Hence, the purpose of this study was to use the multi-theory model (MTM) in predicting initiation and sustenance of plain water consumption instead of sugar-sweetened beverages among college students.

**Study design:** A cross-sectional study.

**Methods:** In this cross-sectional study, a 37-item valid and reliable MTM-based survey was administered to college students in 2016 via Qualtrics at a large public university in the Southeastern United States. Overall, 410 students responded to the survey; of those, 174 were eligible for the study and completed it.

**Results:** Stepwise multiple regression analysis revealed that 61.8% of the variance in the initiation of drinking plain water instead of SSBs was explained by behavioral confidence (*P*<0.001) and changes in the physical environment (*P*<0.001). Further, 58.3% of the variance in the sustenance of drinking plain water instead of SSBs was explained by emotional transformation (*P*<0.001) and practice for change (*P*=0.001).

**Conclusions:** Multi-theory model of health behavior change is a robust theory for predicting plain water consumption instead of SSBs in college students. Interventions should be developed based on this theory for this target population.

## Introduction

Although water intake is vital to human health, many adults do not consume adequate water^1-3^. The problem of insufficient plain water consumption among adults is compounded with the issue of increased consumption of sugar-sweetened beverages (SSBs)^4-7^. SSBs include but are not limited to non-diet carbonated soda, fruit juices, sports drinks, sweetened tea, and energy drinks^8^. Consumption of SSBs is associated with weight gain, obesity, type 2 diabetes mellitus, dental carries, and increased risk for cardiovascular disease^9-12^.

Given that SSB consumption increases from childhood to young adulthood^13^, college years are a critical time to establish healthy beverage consumption. College is a period when many young adults develop healthy behaviors that may continue into adulthood^14^ and often have more autonomy to make nutritional choices^15^, including beverage consumption. Research is needed to identify modifiable factors that influence college students’ initiation and sustenance of plain water consumption instead of SSBs.

A few studies have been conducted to understand beverage consumption patterns or determinants of beverage consumption among university students^6, 7, 16, 17^. Only one investigation has quantitatively assessed theoretical correlates of SSBs consumption among a university population^6^. This study assessed personal and environmental predictors of soda consumption among Belgian university students by measuring constructs from social cognitive theory and the theory of planned behavior^6^. Subjective norm perceived control, self-efficacy, modeling, and family rules predicted college students’ soft drink consumption. No quantitative studies have explored theoretical predictors of plain water consumption among university students in the United States of America.

This study utilized a new behavioral theory called multi-theory model (MTM) of health behavior change^18, 19^. The theory divides behavior change into two components: initiation and sustenance. There are three constructs for initiation of behavior change: 1) participatory dialogue, in which advantages of behavior change must outweigh disadvantages; 2) behavioral confidence, which is the futuristic confidence in one’s ability to perform a behavior change and is sourced not just in the self but may be external to self, such as powerful others, deities, God, etc.; and 3) changes in physical environment such as access, availability, and obtainability of resources that tangibly help with behavior change. For sustenance of behavior change, three constructs have been proposed: 1) emotional transformation, which is the ability to convert feelings into goals for behavior change; 2) practice for change, which entails constantly thinking about behavior change, making mid-term corrections to one’s strategy, overcoming barriers, and remaining focused on health behavior change; and 3) changes in social environment, which includes creating social support that helps with behavior change. The theory has been applied to predicting changes in other obesogenic related behaviors among university student populations including physical activity and changes in portion size^20, 21^.

We tested the MTM in predicting initiation and sustenance of plain water consumption instead of SSBs among U.S. college students. This investigation provides theory-based evidence to assist with the development of interventions to promote plain water consumption instead of SSBs among university students.

## Methods

### Study Design

The study utilized a cross-sectional design. The independent variables were the constructs of the MTM (participatory dialogue, behavioral confidence, changes in physical environment, emotional transformation, practice for change, and changes in social environment) in two models. The two dependent variables for each of the two models were the intent to initiate behavior change with regard to drinking water instead of SSBs and intent to sustain behavior change with regard to drinking water instead of SSBs.

### Population and sample

Students from a large university in the Southeastern United States who reported consuming at least one SSB in the past 24 h from the subject pool for this study. G*Power was used to calculate sample size^22^. The effect size was assumed small to medium at 0.07. The alpha was set at 0.05 and power was 0.80 with three predictors in each model. With these assumptions, the sample size was calculated as 172. The instrument was administered electronically to the university students via Qualtrics in 2016. Two reminder emails were sent. Informed consent was part of the electronic communication in the Qualtrics survey. Since this study was about health behavior change, the survey included a screening question that asked potential participants to recall if they had consumed an SSB in the past 24 h. Only students who had consumed 8 oz. glasses of SSB (e.g. non-diet carbonated soda, fruit juice sports drinks, sweetened tea, energy drinks, etc.) in the past 24 h were included in the study.

The University of Mississippi Institutional Review Board (IRB) permission was obtained prior to conducting the study.

### Measures and instrument

The instrument used in this study consisted of 37 items. Informed consent was the first part of the instrument. The instrument was developed by the first author and validated for face and content validity in a two-round process by a panel of six experts. Eight experts were contacted via email to serve on the panel, of whom six agreed to participate. Five of the experts were university professors and one was a health educator in a university setting. Therefore, all of the experts had firsthand understanding of college students (target population). Five of the experts were instrumentation experts having developed theory-based questionnaires themselves in the past. One of them had previously published on MTM and one had familiarity with MTM. Constitutive and operational definitions of all constructs with scoring key were provided to all experts. In the first round, the panel of experts contributed 41 comments that pertained to sentence construction, readability, and formatting. All of the comments were addressed and consensus was achieved at the end of the second round where the panel of experts agreed that the instrument was ready for administration. The final instrument had a Flesch-Kincaid Grade level of 5.4 and a Flesch Reading Ease score of 66.9, indicating that it was easily readable for the priority population. Cronbach’s alpha was computed to establish internal consistency of the subscales and the scale.

The first item on the instrument after the consent was a screening question. The next seven questions were about demographics including gender, age, race/ethnicity, class, current overall GPA, place of dwelling, and work status. Advantages of drinking water instead of SSBs on the instrument included being healthy, not feeling lethargic, managing weight, having more energy, and saving money. This construct was measured by items 9-13 as a summative score on a scale of never (0), hardly ever (1), sometimes (2), almost always (3), and always (4), yielding a possible range of 0-20. Disadvantages of drinking plain water instead of SSBs included having less enjoyment, being hungry most of the time, having less energy, missing carbonation, and missing caffeine. This construct was measured by items 14-18 as a summative score on a scale of never (0), hardly ever (1), sometimes (2), almost always (3), and always (4), yielding a possible range of 0-20. Behavioral confidence was reified as surety of drinking water instead of SSBs this week, while eating out, while watching TV or sports etc., without missing caffeine, and without missing carbonation. This construct was measured by items 19-23 as a summative score on a scale of not at all sure (0), slightly sure (1), moderately sure (2), very sure (3), and completely sure (4), yielding a possible range of 0-20. Changes in physical environment were reified as surety for being able to eliminate SSBs from one’s physical environment, being able to drink water instead of SSBs when others around one are drinking those, and being able to buy water instead of SSBs when needed. This construct was measured by items 24-26 as a summative score on a scale of not at all sure (0), slightly sure (1), moderately sure (2), very sure (3), and completely sure (4), yielding a possible range of 0-12.

The first construct in the sustenance model was emotional transformation reified as surety of directing feelings/emotions, motivating oneself, and overcoming self-doubt to drink plain water instead of SSBs every day. This construct was measured by items 27-29 as a summative score on a scale of not at all sure (0), slightly sure (1), moderately sure (2), very sure (3), and completely sure (4), yielding a possible range of 0-12. The second construct in the sustenance model was practice for change reified as surety of keeping a self-diary, overcoming barriers, and rectifying one’s plan to drink plain water instead of SSBs every day. This construct was measured by items 30-32 as a summative score on a scale of not at all sure (0), slightly sure (1), moderately sure (2), very sure (3), and completely sure (4), yielding a possible range of 0-12. The final construct in the sustenance model was changes in social environment and was reified as surety of soliciting the help of family member (s), friend (s), and/or health professional (s) to drink plain water instead of sugar-sweetened beverages every day. This construct was measured by items 33-35 as a summative score on a scale of not at all sure (0), slightly sure (1), moderately sure (2), very sure (3), and completely sure (4), yielding a possible range of 0-12.

Initiation of drinking water instead of SSBs was defined as the likelihood of a person to drink plain water instead of SSBs every day in the upcoming week. This was measured by one item using a Likert-type scale of not at all likely (0), somewhat likely (1), moderately likely (2), very likely (3), and completely likely (4) with a possible range of 0-4. Likewise, sustenance of drinking plain water instead of SSBs was defined as the likelihood of a person to drink plain water instead of SSBs every day from now on. This was measured by one item using a Likert-type scale of not at all likely (0), somewhat likely (1), moderately likely (2), very likely (3), and completely likely (4) with a possible range of 0-4.

### Statistical Analyses

All data were analyzed using SPSS, version 21 (Chicago, IL, USA). For descriptive statistics, means, standard deviations of metric variables, and frequencies and percentages for categorical variables were reported. With a sample size of 174 subjects and a large number of parameters in our model, SEM estimates are not stable^23^. Thus, we chose to use stepwise multiple regressions to build the initiation and sustenance models. For stepwise multiple regression, the a priori criteria of probability of F to enter the predictor in the model was set as less than or equal to 0.05 and for removing the predictor as greater than or equal to 0.10.

## Results

Totally, 410 respondents attempted the survey of which 174 met the inclusion criteria and completed the survey. Descriptive statistics for the demographic variables are presented in Table 1. The mean age of the respondents was 23.82 yr (S.D.=7.58) and there were more female (65.5%) respondents. A majority of the respondents was white (79.9%), lived off-campus (78.6%), and was employed (55.5%).

**Table 1:** Socio-demographic characteristics of the study participants (n = 174)

**Characteristics**	**Number**	**Percent**
Gender		
Male	60	34.5
Female	114	65.5
Race/Ethnicity		
White/Caucasian	139	79.8
African American	20	11.4
Asian American	4	2.3
Hispanic American	5	2.8
Other	6	3.4
Class Level		
Freshmen	23	13.3
Sophomore	31	17.9
Junior	35	20.2
Senior	41	23.7
Graduate	43	24.9
Current overall GPA		
≤1.99	2	1.2
2.00 - 2.49	10	5.8
2.50 - 2.99	28	16.2
3.00 - 3.49	56	32.4
3.50 - 4.00	77	44.5
Living Arrangements		
On campus	37	21.4
Off-campus	136	78.6
Work status		
Yes	96	55.5
No	77	44.5

The means and standard deviations of study variables and corresponding Cronbach’s alphas are depicted in Table 2. All the Cronbach’s alphas are over 0.70 thus indicative of internal consistency of the subscales^24^. The mean score of participatory dialogue was 9.20 units out of a possible –20-+20, indicating that respondents were already perceived the advantages to outweigh disadvantages of behavior change from consuming SSBs to plain water. Likewise, the constructs of behavioral confidence (M=11.05) and changes in physical environment (M=7.65) were in the middle of the possible range. The constructs of sustenance, namely emotional transformation (M=8.33), practice for change (M=6.28) and changes in social environment (M=7.96) were also at or slightly above the middle of the possible range.

**Table 2:** Descriptive statistics of study variables (n=174)

**Constructs**	**Possible Range**		**Observed Range**		**Mean**	**SD**	**Cronbach’s** **alpha**
	**Min**	**Max**	**Min**	**Max**			
Participatory dialogue							
Advantages	0	20	0	20	16.12	3.21	0.79
Disadvantages	0	20	0	20	7.00	3.86	0.75
Advantages - disadvantages score	-20	20	-16	20	9.20	5.68	---
Initiation	0	4	0	4	2.79	1.32	---
Behavioral confidence	0	20	0	20	11.05	5.38	0.85
Changes in physical environment	0	12	0	12	7.65	3.23	0.79
Sustenance	0	4	0	4	2.56	1.33	---
Emotional transformation	0	12	0	12	8.33	3.43	0.95
Practice for change	0	12	0	12	6.28	3.17	0.78
Changes in social environment	0	12	0	12	7.96	3.34	0.79
Entire scale	---	---	---	---	---	---	0.90

Results of stepwise multiple regression analysis for the initiation model of drinking plain water instead of SSBs are depicted in Table 3. The analysis revealed that 61.8% of the variance in the initiation of drinking plain water instead of SSBs was explained by behavioral confidence and changes in physical environment, *F* (2, 173) = 142.6, *P*<0.001. Results of sustenance of drinking plain water instead of SSBs model are depicted in Table 4. 58.3% of the variance in the sustenance of drinking water instead of SSBs was explained by emotional transformation and practice for change, *F* (2, 172)=122.594, *P*<0.001.

**Table 3:** Parameter estimates based on stepwise regression analysis to predict initiation of plain water consumption behavior change (*n*=174)

**Variables**	**B**	**SE**_B_	**β**	**95% CI**	***P *** **value**
Behavioral confidence	0.105	0.017	0.426	0.071, 0.139	0.001
Changes in physical environment	0.170	0.029	0.417	0.113, 0.226	0.001

*F*(2, 173) = 142.627, *P*< 0.001, *R*^2^(Adjusted *R*^2^) = 0.622 (0.618) Dependent variable is initiation of water consumption behavior change; B = unstandardized coefficient; SE_B_= standard error of the coefficient; β = standardized coefficient

**Table 4:** Parameter estimates based on stepwise regression analysis to predict sustenance of plain water consumption behavior change (n=173)

**Variables**	**B**	**SE**_B_	**β**	**95% CI**	***P *** **value**
Emotional transformation	0.210	0.030	0.549	0.151, 0.269	0.001
Practice for change	0.107	0.032	0.257	0.043, 0.171	0.001

F(2, 172) = 122.594, *P* < 0.001, R^2^(Adjusted R^2^) = 0.588 (0.583) Dependent variable is sustenance of water consumption behavior change; B = unstandardized coefficient; SE_B_= standard error of the coefficient; β = standardized coefficient

## Discussion

The purpose of this study was to apply the MTM of health behavior change to predict initiation and sustenance of plain water consumption behavior instead of consuming SSBs in a sample of university students. For initiation of plain water consumption behavior instead of consuming SSBs, the constructs of behavioral confidence (*P*<0.001, and changes in physical environment (*P*<0.001) predicted almost 62% of the variance, which is considerably high for behavioral studies. The results are similar to a study with Belgian university students that also found significant predictive value for self-efficacy and perceived behavioral control in lowering SSBs^6^. Self-efficacy and perceived behavioral control were related to the MTM construct of behavioral confidence in lowering consumption of SSBs^6^. Likewise, the study tested the role of physical environment in predicting SSB consumption using the variables of perceived availability and distance to stores, and also found these variables to be significant^6^. The current study underscores the importance of the two constructs of behavioral confidence and changes in physical environment in helping start the behavior change of plain water consumption instead of SSBs. The construct of participatory dialogue, in which advantages of behavior change of water consumption of SSB outweigh the disadvantages of the behavior change, was not significant. This could be due to the overall score of participatory dialogue (M=9.20), which was already high indicating that respondents were already convinced that drinking plain water was good for them and so it did not further play any role in the decision-making process.

For sustenance of plain water consumption behavior instead of consuming SSBs, the constructs of emotional transformation (*P*<0.001) and practice for change (*P*<0.001) predicted almost 58% of the variance which is sizable high for behavioral studies. These constructs have not been studied in previous literature with regard to this behavior. However, the construct of social environment has been studied and found to be predictive of low SSB consumption in college students^6^. We did not find the role of social environment as predictive of sustenance of plain water consumption behavior instead of consuming SSBs in the current study. Perhaps future studies need to examine this construct more carefully.

Overall, 410 respondents attempted the survey; of those, 174 met the inclusion criteria and completed the survey. This implies that 236 (57.6%) respondents did not consume an SSB in the past 24 h. This reflects growing awareness in university students about the harmful effects of SSBs; consequently, a large proportion of university students appear to be abstaining from SSB consumption. Conversely, there is also a substantial proportion (42.4%) of college students who engage in this behavior and there is a need to design educational interventions for this group of students.

This study had some limitations. First, the sampling method chosen for this study was a quota sample and not a random sample. This limits the generalizability of the findings to the entire population of college students. However, sample size estimation was conducted therefore, the choice of a quota sample is justifiable. Future studies employing the MTM should utilize random sampling. Second, the research design chosen for the study was cross-sectional. These designs cannot provide evidence for cause-effect relationships because all the variables are measured at the same point in time. However, the constructs precede the behavior so we can fall back on the theory to justify the design. Future studies should consider utilizing longitudinal designs to examine the temporal relationship of the MTM constructs. Finally, there were some deficiencies in measurement such as use of self-report, not testing for construct validity, and not testing for stability reliability. No other method can be used other than self-report when measuring theoretical constructs; however, observation or blood biochemistry can be employed to measure behaviors. Future studies should consider testing construct validity, conducting test-retest reliability, and employing more valid methods valid to improve measurement of behaviors.

There is a definitive need to design interventions that promote plain water consumption instead of consuming SSBs for university students. In order to initiate this behavior change in students already habituated to drinking SSBs, behavioral confidence to make this switch is of paramount importance. In order to build behavioral confidence, the steps for switching can be broken down into small steps. For example, practitioners can encourage students to have a visual record of how much water they should consume daily. Effective techniques such as role-play, simulation or psychodrama can also be employed. Sources of behavioral confidence such as self-confidence, powerful others, faith in deities; faith in God, etc. can be mobilized. The other construct that needs to be modified for initiation of plain water consumption behavior instead of consuming SSBs is changing in physical environment. In order to influence the construct of elimination of SSBs from one’s physical environment, being able to drink plain water instead of sweetened beverages when others around one are drinking those, and being able to obtain plain water instead of SSBs when needed are some important steps.

In order to sustain plain water consumption behavior instead of consuming SSBs the first construct that needs to be modified is that of emotional transformation. In order to influence this construct, one needs to be cognizant of one’s emotions and then whenever one recognizes these emotions one must transform these into behavioral goals of changing the behavior from drinking SSBs to plain water. The other construct that needs to be influenced is that of practice for change. Techniques such as keeping a personal journal, identifying and overcoming barriers, and rectifying one’s plan to drink water instead of SSBs every day. These measures can be instituted in one-on-one counseling programs, group programs, or campus-wide initiatives.

MTM of health behavior change is an emerging theory and more studies with a multitude of health behaviors need to be undertaken. Studies should include both descriptive predictive studies in behavioral epidemiology like this study, as well as, interventional studies using randomized controlled designs (RCTs) or even quasi-experimental and pre-experimental designs. Such empirical research will enrich the field of health behavior, health education, and health promotion practice and research.

## Conclusions

MTM of health behavior change appears to be a robust theory for both predicting initiation and sustenance of plain water consumption behavior instead of consuming SSBs among university students. Effective interventions based on this theory should be developed for this target population.

## Acknowledgements

The authors would like to thank Jackson State University, University of North Carolina at Wilmington, Lincoln Memorial University and University of Mississippi for their support in completion of this study.

## Conflict of interest statement

The authors declare that there is no conflict of interest.

## Funding

None.

## Highlights

- Behavioral confidence and changes in physical environment significantly predicted initiation of drinking plain water.
- Emotional transformation and practice for change significantly predicted sustenance of drinking plain water.
- MTM is a robust theoretical model for the prediction of drinking plain water behavior change.
